# Conducting invasive urodynamics in primary care: qualitative interview study examining experiences of patients and healthcare professionals

**DOI:** 10.1186/s41512-021-00100-y

**Published:** 2021-05-18

**Authors:** Sarah Milosevic, Natalie Joseph-Williams, Bethan Pell, Elizabeth Cain, Robyn Hackett, Ffion Murdoch, Haroon Ahmed, A. Joy Allen, Alison Bray, Samantha Clarke, Marcus J. Drake, Michael Drinnan, Kerenza Hood, Tom Schatzberger, Yemisi Takwoingi, Emma Thomas-Jones, Raymond White, Adrian Edwards, Chris Harding

**Affiliations:** 1 Centre for Trials Research, Cardiff University, Cardiff, UK; 2 PRIME Centre Wales, Division of Population Medicine, School of Medicine, Neuadd Meirionnydd, Cardiff University, Cardiff, UK; 3 Centre for the Development and Evaluation of Complex Interventions for Public Health Improvement (DECIPHer), Cardiff University, Cardiff, UK; 4 NIHR Newcastle In Vitro Diagnostics Co-operative, Newcastle University, Newcastle upon Tyne, UK; 5 Translational and Clinical Research Institute, Newcastle University, Newcastle upon Tyne, UK; 6 Northern Medical Physics and Clinical Engineering, The Newcastle upon Tyne Hospitals NHS Foundation Trust, Royal Victoria Infirmary, Newcastle upon Tyne, UK; 7 North Bristol NHS Trust, Southmead Hospital, Southmead Road, Westbury-on-Trym, Bristol, UK; 8 Translational Health Sciences, Bristol Medical School, University of Bristol, Bristol, UK; 9 Corbridge Health Centre, NHS Northumberland Clinical Commissioning Group, Newcastle Road, Corbridge, Northumberland, UK; 10 Test Evaluation Research Group, Institute of Applied Health Research, University of Birmingham, Birmingham, UK; 11 PPI Representative, formerly of Grampian University Hospital Trust, Biomedical Physics and Bioengineering, Foresterhill, Aberdeen, UK; 12 Department of Urology, Newcastle upon Tyne NHS Hospital Trust, Newcastle Freeman Hospital, Freeman Road, High Heaton, Newcastle upon Tyne, UK

**Keywords:** Urodynamics, Primary care, Lower urinary tract symptoms, Qualitative research

## Abstract

**Background:**

Invasive urodynamics is used to investigate the causes of lower urinary tract symptoms; a procedure usually conducted in secondary care by specialist practitioners. No study has yet investigated the feasibility of carrying out this procedure in a non-specialist setting. Therefore, the aim of this study was to explore, using qualitative methodology, the feasibility and acceptability of conducting invasive urodynamic testing in primary care.

**Methods:**

Semi-structured interviews were conducted during the pilot phase of the PriMUS study, in which men experiencing bothersome lower urinary tract symptoms underwent invasive urodynamic testing along with a series of simple index tests in a primary care setting. Interviewees were 25 patients invited to take part in the PriMUS study and 18 healthcare professionals involved in study delivery. Interviews were audio-recorded, transcribed verbatim and analysed using a framework approach.

**Results:**

Patients generally found the urodynamic procedure acceptable and valued the primary care setting due to its increased accessibility and familiarity. Despite some logistical issues, facilitating invasive urodynamic testing in primary care was also a positive experience for urodynamic nurses. Initial issues with general practitioners receiving and utilising the results of urodynamic testing may have limited the potential benefit to some patients. Effective approaches to study recruitment included emphasising the benefits of the urodynamic test and maintaining contact with potential participants by telephone. Patients’ relationship with their general practitioner was an important influence on study participation.

**Conclusions:**

Conducting invasive urodynamics in primary care is feasible and acceptable and has the potential to benefit patients. Facilitating study procedures in a familiar primary care setting can impact positively on research recruitment. However, it is vital that there is a support network for urodynamic nurses and expertise available to help interpret urodynamic results.

## Background

Lower urinary tract symptoms (LUTS) are highly prevalent amongst men aged over 40, with over 70% reporting at least one symptom [1], and are associated with reduced emotional wellbeing, productivity, and quality of life [2, 3]. Urodynamics is a specialist test for investigating the causes of LUTS [4]. It is typically conducted in secondary care and involves inserting catheters into the bladder and rectum to enable measurements pertaining to bladder function. A growing number of patients presenting with LUTS are eventually treated conservatively and thus could potentially be effectively managed in primary care [5, 6]. Managing LUTS in primary care settings could result in benefits for the patient and cost benefits for the NHS. However, general practitioners (GPs) currently do not have access to simple tools to accurately diagnose the cause of LUTS in men.

Recognising this issue, the UK National Institute for Health Research (NIHR) released a Health Technology Assessment (HTA) commissioned call for the development of a clinical decision aid to help inform treatment choice or need for specialist referral for men presenting with LUTS in primary care. The PriMUS study (‘Primary care management of lower urinary tract symptoms in men: development and validation of a diagnostic and clinical decision support tool’) addresses this brief [7]. PriMUS is a prospective diagnostic accuracy study based in UK primary care settings. Participants undergo simple index tests together with invasive urodynamics as a reference standard, to determine the combination of index tests that best predicts common urodynamic diagnoses. This will inform the development of a clinical decision support tool for use by GPs with their patients.

Invasive urodynamics is a complex procedure with risk of adverse effects, yet very few qualitative studies have explored patient acceptability of this test. One interview study [8] found patients experienced anxiety and embarrassment about the procedure, which was alleviated by healthcare professionals through effective interpersonal and communication skills. Previously, men participating in a randomised controlled trial involving urodynamics [9] found the procedure acceptable and valued the comprehensive insight into their symptoms. As healthcare professionals have been found to play an important role in the patient experience of urodynamics [8, 9], it is important to further explore their perspectives. Furthermore, no study has explored in-depth the attitudes of patients who have declined urodynamics, who may view the procedure differently [9].

PriMUS is the first large-scale study to implement invasive urodynamics in a primary care setting; no study has yet investigated the feasibility and acceptability of conducting this procedure in a non-specialist setting. This study aimed to explore in-depth the feasibility and acceptability of providing invasive urodynamics in primary care, including experiences of recruiting to a urodynamic study, encompassing the perspectives of patients (including those who declined the procedure) and healthcare professionals. Including these participant groups provided further insight into attitudes towards and experiences of urodynamics, and the feasibility of providing urodynamics in primary care, and sought to inform processes or interventions that could improve acceptability of this invasive procedure. Given the exploratory nature of the research, a qualitative approach was needed to enable detailed insight into participant experiences that could not be obtained via quantitative methods such as questionnaires. The qualitative study was conducted during the pilot phase of the PriMUS study, so that findings could inform changes to study processes where indicated.

## Methods

### Study design

This qualitative study was part of the larger PriMUS study, which gained ethical approval from Wales Research Ethics Committee 6. PriMUS is recruiting adult men presenting to their GP with one or more bothersome LUTS, in approximately 90 GP practices across Newcastle upon Tyne, South Wales and Bristol (see study protocol [7] for full inclusion and exclusion criteria). Participants to date underwent a series of index tests together with invasive urodynamics, which is usually carried out in secondary care by specialist practitioners. In the PriMUS study, the procedure was performed by trained research nurses in primary care settings. Results of the urodynamic procedure and index tests were uploaded to an online database, quality-checked and reviewed by clinical scientists and urologists on the study team (AB, MD, CH and MJD), and a summary of results for GPs was compiled manually. This included the likely diagnosis(es) and a flowchart of management recommendations based on the National Institute for Health and Care Excellence (NICE) guidelines [10].

Before conducting urodynamics as part of the study, research nurses were provided with a urodynamics manual and underwent a series of training activities to ensure competency, including completing an accredited urodynamic training course overseen by study urologists, shadowing urologists in secondary care settings and completing study specific interpretation and equipment training. They receive frequent, ongoing peer and clinical support from members of the study management team and have access to a named Urology Champion in each study region.

Qualitative methods such as interviews are particularly appropriate to increase understanding of patient experiences of trial processes [11] and can allow for the exploration of previously unanticipated issues that may be missed in quantitative studies. Therefore, semi-structured telephone interviews were conducted with men invited to take part in the main PriMUS study. Telephone and face-to-face interviews were also carried out with healthcare professionals involved in study delivery, including GPs and practice nurses involved in study recruitment, and research nurses who performed the urodynamic procedure.

### Sampling and recruitment

Purposive sampling was used to support maximum variation in terms of study site, decision to participate in the main PriMUS study (for patients) and role in study delivery (for healthcare professionals). Patients invited to participate in the main study could indicate whether they consented to be contacted for a follow-up interview; this included both patients who consented to the main study and those who declined to participate. Patients consenting to be contacted were invited to take part in an interview once they had undergone all study procedures or had made the decision not to participate in the main PriMUS study. Interview participants were offered a £10 voucher for their contribution. Healthcare professionals were approached to take part once they had experience of study processes (e.g. recruiting patients or performing urodynamics). Recruitment continued until data saturation was reached. Informed consent (written or verbal) was obtained for all face-to-face and telephone interviews after participants had sufficient time to read the interview study information sheet.

### Data collection

Semi-structured interview topic guides were developed in consultation with clinicians and patient representatives on the study team. Topics explored in patient interviews included thoughts about the main study information, the decision of whether to take part, concerns about the study and how others could be encouraged to participate. Those participating in the main study were also asked about their experience of study processes and specifically the urodynamic test. Topics covered in healthcare professional interviews included (as applicable) experiences of study recruitment, perceived patient acceptability of study processes, staff experiences of performing the urodynamic test and the feasibility of doing so in primary care. Topic guides were developed iteratively throughout the data collection period to allow for the exploration of previously unanticipated themes arising from the interviews. For example, after issues relating to urodynamic test results were highlighted by GPs, this was specifically explored in subsequent interviews.

Interviews were conducted between May 2018 and February 2019. Most were conducted by SM, an experienced qualitative health researcher, with the remainder conducted by medical students (EC, RH and FM) supervised by SM. To encourage respondents to give honest, unbiased feedback about their experience of the main study, the interviewers were not previously known to participants and had no involvement in main study procedures. All interviews were audio-recorded and transcribed verbatim for analysis.

### Analysis

A framework approach [12] was used to analyse interview data. SM compiled a list of key categories to explore, with reference to the interview topic guide: (1) acceptability of invasive urodynamics in primary care, (2) feasibility of invasive urodynamics in primary care and (3) experiences of recruiting to a urodynamic study. After reading through transcripts, emergent sub-themes were identified within these three categories in the initial framework. Analysis was an iterative process, enabling interviews to continue until saturation was reached. Saturation was determined as being achieved once interviews resulted in no new themes and when detailed data had been collected relating to each identified theme. SM entered a framework of categories and sub-themes into NVivo V11 (QSR International), then uploaded and coded interview transcripts. Ten percent of transcripts was independently coded by NJW, and coding was compared and discussed to ensure inter-rater consistency. Sub-themes were refined throughout the coding process to ensure they captured the diversity of participants’ experiences. Once all interviews were coded, SM compiled tables to show a summary of each participant’s responses in relation to each category and sub-theme. Tables included data extracts from the coded interview transcripts; this enabled the validity of the proposed themes to be reviewed by SM and NJW, ensuring that there were sufficient data to support each of the emergent sub-themes, and that they provided an accurate reflection of participant experiences.

## Results

Interviews were conducted with 25 male patients and 18 healthcare professionals, from 22 GP practices across Newcastle upon Tyne, South Wales and Bristol (Table 1). Interviews with patients lasted between 8 and 44 min (mean 23.0); interviews with healthcare professionals lasted between 9 and 30 min (mean 18.8). Eight sub-themes were identified under the three main framework categories: Acceptability of invasive urodynamics in primary care (three sub-themes), feasibility of invasive urodynamics in primary care (three sub-themes) and recruiting to a urodynamic study (two sub-themes) (see Table 2). Participant quotes are labelled with a unique participant identification number, with the prefix MSP for patients who participated in the main study, IP for patients who participated in the interview element only, GP for general practitioners and UN for urodynamic nurses.

**Table 1:** Participant characteristics

Patients (***n*** = 25)		Healthcare professionals (***n*** = 18)	
	***N***		***N***
**Participant in main study**		**Role**	
Yes	22	GP	11
No	3	Practice nurse	3
		Urodynamic nurse	4
**Geographical region**		**Geographical region**	
Newcastle upon Tyne	10	Newcastle upon Tyne	6
South Wales	9	South Wales	7
Bristol	6	Bristol	5
**Age group**^a^			
46–55	3		
56–65	6		
66–75	8		
76–85	5		

^a^Participant age was not recorded for patients who did not participate in the main PriMUS study

**Table 2:** Key framework categories and emergent sub-themes

Key framework categories	Emergent sub-themes
Acceptability of invasive urodynamics in primary care	Apprehension and embarrassment
	Communication
	Preference for primary care
Feasibility of invasive urodynamics in primary care	Training and support
	Logistical issues
	Difficulties receiving and using results
Recruiting to a urodynamic study	Importance of proactive recruitment
	Reasons for participation and non-participation

### Acceptability of invasive urodynamics in primary care

#### Apprehension and embarrassment

All patients who underwent urodynamics reported finding the procedure acceptable, with most finding it as they had expected, or better than expected. Around half reported discomfort, although this was universally described as brief or mild. Some patients described feeling apprehensive about the test; those with experience of similar medical procedures (e.g. cystoscopy) reported lower anxiety. The invasive nature of the procedure was mentioned by several patients; some commented that this was not an issue for them, while others found it embarrassing, particularly where the procedure was conducted by female healthcare professionals. While one suggested this was due to his older age, another explained how he accepted that getting older meant invasive procedures were more likely (Table 3).

**Table 3:** Acceptability of invasive urodynamics in primary care

**Apprehension and embarrassment: **	
*It was a little bit… uncomfortable at first, but I mean not greatly so… It was only two seconds… I was a little bit… nervous because I didn’t know how painful it was going to be. But it was actually… nothing like what I thought it was going to be you know. It was… a lot nicer or better… (MSP 2015, age 65)*	
*She explained it was going to feel a bit uncomfortable when they did it, and it did, it tingled a bit but that was about it, it was very much as I expected. (MSP 1023, age 73)*	
*No concerns at all really… I’d had that camera or catheter, or whatever you call it, in my bladder twice before. So, I, it didn’t put me off at all. (MSP 3103, age 74)*	
*My biggest problem … I did feel embarrassed with some of the procedure, you know…* [*I’m*] *old fashioned, that’s the trouble. (MSP 1117, age 84)*	
*If you’re male and you’ve got three women… you know it’s a bit embarrassing… I just convinced myself like you’re an old man now, you know, they see these things all the time. So I managed to rationalise that… and I realise as I get older you might have to put up with more of that stuff. (MSP 1025, age 57)*	

*MSP* main study participant

#### Communication

A key factor in patient acceptability was the extent to which nurses explained the test, supported patients with information provision in advance and through the procedure and made them feel at ease. Patients reported they had been given the right level of information, so understood the purpose of the test, and that the nurses made the procedure as comfortable as possible, which reduced their anxiety. The ‘respectful’ and ‘professional’ manner of nurses in discussing and conducting urodynamics also helped to reduce patient embarrassment.

#### Preference for primary care

Undergoing the urodynamic test in primary rather than secondary care was viewed positively by patients, mainly due to convenience (e.g. reduced waiting and travel time and ease of parking) and familiarity with surgery staff. Several patients explained they had ‘full confidence’ in their GP practice and felt relaxed about visiting the surgery, while they would be more apprehensive if they had to attend hospital for the same procedure (Table 4). Some suggested that they would not have agreed to undergo urodynamics if the test had been conducted in hospital.

**Table 4:** Acceptability of invasive urodynamics in primary care

**Communication:**	
*They explained everything well… I thought it was quite an uncomfortable test and quite deep. But… they were very professional, the way they talked about it and dealt with it… they were friendly, they spoke to you during it, you know, you weren’t just like lay there… they tried to make you as comfortable as possible. (MSP 2002, age 55)*	
*I found it quite easy, really, with the people I saw, you know, all the way through… That was nice not to have to worry about the people… you knew that they were explaining it to you and helping you, and guiding you, really. (MSP 3103, age 74)*	
*I was a bit worried at the start… but the girls that did it were, made you feel at ease and were brilliant, so I didn’t really feel under any pressure. (MSP 3110, age 56)*	
**Preference for primary care:**	
*I* [*can’t*] *see any benefit* [*to having the test done in hospital*]*, I think it was better at my doctors, it was a lot more relaxed, because obviously I knew the nurse and I knew the doctor and you know, I had chatted with them about the test. (MSP 2002, age 55)*	
*It saved me going to hospital and… getting a taxi and one thing and another. I think that’s more why I did it sort of thing I think at the doctors rather than traipsing round the country. (MSP 3110, age 56)*	
*It’s more personal with the doctor. And he knows your history and that. (MSP 2006, age 77)*	

*MSP* main study participant

### Feasibility of invasive urodynamics in primary care

#### Training and support

Urodynamic nurses reported that facilitating urodynamics in primary care was generally a positive experience. Most had not independently performed invasive urodynamics before receiving training for the PriMUS study and were apprehensive about conducting the procedure in a community setting, but all felt their confidence grew as they gained experience. They valued the initial training and ongoing support provided by the study team, particularly regular teleconferences with peers and access to ad-hoc telephone advice.

#### Logistical issues

Several logistical issues related to performing urodynamics in primary care were identified. Facilities at GP practices varied, and the procedure was sometimes carried out in unsuitable rooms, for example with a carpeted floor that was difficult to clean, insufficient space or difficulty accessing a sluice. Remote working meant nurses had to transport heavy equipment and sometimes experienced internet connectivity issues, which had led to clinic cancellations.

#### Difficulties receiving and using results

GPs highlighted problems with receiving and utilising urodynamic results in the initial stages of the study. Study-specific quality assurance procedures meant GP summaries took longer to be returned than expected, and where concurrent diagnoses were identified, GPs were unsure which to treat first. It was suggested this limited the potential benefit of the study to some patients (Table 5). As GP interviews were conducted in the pilot phase of the PriMUS study, their feedback enabled changes to be made to site training and the process of obtaining results.

**Table 5:** Feasibility of invasive urodynamics in primary care

**Training and support:**	
*It was difficult to begin with and I like being challenged so that was interesting… Being able to go to the… urodynamics course was fantastic, that was really, well essential but really, really interesting and useful as well and the peer support that we get* [*from*] *the fortnightly nurse teleconferences* [*was*] *particularly useful at the beginning. (UN 506)*	
*We had issues on our very first patient and we were lucky that we’re able to phone up… and say what shall we do about this… Having someone on the end of the line is reassuring. (UN 607)*	
**Logistical issues:**	
[*The equipment is*] *heavy. It’s really bulky. And it’s sensitive equipment as well… It’s been hard graft. You’ve got to get it all in the car, get it all out… up hills and over steps and in to little rooms. So it’s not good for the equipment. It’s not really good for my back* [*laughs*]*… It’s quite stressful… making sure I’ve got every single thing to run the test. (UN 606)*	
*We don’t always have the most appropriate room available to us in the GP surgeries… Sometimes the rooms are tiny, and you literally are falling over each other… sometimes the electricity sockets are not in the right place, so we have to use our extension lead… there’s lots of sort of improvisation… I think we manage it quite well, but it is a challenge. (UN 607)*	
**Difficulties receiving and using results:**	
*You tell* [*patients that urodynamics*] *is the gold standard of investigation… And that it could help further… define what the problem is, and… target treatment a bit better… However… I’ve never had to look at… urodynamics or reports before. And therefore I feel what they’re actually getting out of it is… some half-hearted interpretation of… what might be the best management plan. So although they may be getting gold standard investigation, they’re not necessarily getting gold standard advice. (GP 604)*	

*UN* urodynamic nurse, *GP* general practitioner

### Recruiting to a urodynamic study

#### Importance of proactive recruitment

Main study recruitment was opportunistic or via primary care database searches, with most GP practices using a combination of these approaches. Healthcare professionals emphasised the importance of having a recruitment lead at each practice, to ensure database searches were carried out regularly and to remind others to recruit opportunistically. Maintaining telephone contact with patients invited to take part was highlighted as particularly effective in improving study recruitment and retention.

#### Reasons for participation and non-participation

When explaining the study to patients, clinicians emphasised they would have quicker access to a comprehensive diagnostic test not normally available in primary care, that could help in the diagnosis and treatment of their LUTS. Accordingly, most patients identified this as a key factor in their decision to take part. Another common reason for participation was the altruistic opportunity to contribute to research with the potential to improve medical practice and benefit others in the future. Some participants specifically desired to raise the profile of LUTS in men. Others wanted to participate to help their own GP surgery, particularly where they had built up a good relationship with their GP. Conversely, one patient who declined to take part in the main study explained he was not familiar with any of the GPs at his surgery. For patients who opted not to take part in the study, this was mainly because they did not want to have an invasive test. Those who participated appreciated that the research could be carried out at a local GP surgery, while those who declined generally mistakenly believed they would have to attend hospital (Table 6).

**Table 6:** Recruiting to a urodynamic study

**Importance of proactive recruitment:**	
*If you phone* [*the patient*] *a day or two beforehand, they’re more likely to come in for* [*their study appointment*] … *Because, yeah, a couple of people have said, you know if you didn’t phone I don’t think I’d be here. (UN 606)*	
*Really you need somebody… leading the recruitment… making sure that that database search is done, the list is checked, and the appropriate letter sent out… Which… might slip off people’s radar a little bit if it’s not something that’s done regularly. (GP 401)*	
**Reasons for participation and non-participation:**	
*I should benefit from it and so should other people. So to me, even though it was a little bit intrusive the test, I still think it was for the right reasons… just the knowledge that someone else may benefit from it, makes you feel better. (MSP 2002, age 55)*	
[*The doctor*] *said… the surgery* [*were*] *participating in the PriMUS study and would I be prepared to take part and I said yes because he’s a fabulous doctor. (MSP 1117, age 84)*	
*I said yes, because it was at the surgery, had it… been at the hospital, I would have said oh no… you’re talking an hour, an hour and a half one way… but because it was round the corner to my house, it was totally different. (MSP 2002, age 55)*	
*I don’t like people prodding and poking around my private parts or anything* [*laughs*]*… I thought no way am I letting them mess around with me… because it’s not a very sort of, what shall we say, palatable thing is it really? (IP 301)*	
*I didn’t want* *[to do anything that*] *involved having to go to hospital… I would prefer not to go to hospital… to do any testing at all… If the GP wanted to do it, yes I don’t mind that. (IP 201)*	

*UN* urodynamic nurse, *GP* general practitioner, *MSP* main study participant, *IP* interview-only participant

To improve study recruitment, patients suggested clinicians should emphasise that participants would gain prompt access to a thorough assessment of their symptoms without needing to go to hospital. Additional reassurance about the urodynamic test was recommended; for example, explaining that most patients who experience the test find it acceptable, or giving a detailed timeline of the procedure to show that discomfort associated with catheter insertion is short-lived. Other suggestions included advertising the research more widely, and particularly having the study recommended by a familiar GP. Patients believed those who were uncomfortable with the thought of the urodynamic test would not take part in any instance.

## Discussion

To the best of our knowledge, this is the first study to explore the feasibility and acceptability of invasive urodynamics in primary care, and the first qualitative study of urodynamics to include the perspectives of primary healthcare professionals. Overall, patients found the urodynamic test acceptable; a key factor in this was the extent to which nurses made them feel well prepared and at ease, highlighting the importance of good communication and the patient-clinician relationship. Facilitating urodynamics in a primary care setting benefitted patients, who valued the convenience and familiarity of their GP practice. Urodynamic nurses also found this to be a positive experience, despite the logistical issues associated with remote working, and felt well supported by the study team. However, initial issues with receiving and utilising the results of urodynamic testing may have limited the potential benefit of the procedure to some patients.

Approaches found to be effective in recruiting patients to the urodynamic study included having a recruitment lead at each GP practice, maintaining telephone contact with potential participants and emphasising to patients that taking part would give them quicker access to a thorough diagnostic test. Patients’ relationship with their GP was an important influence on study recruitment; performing invasive urodynamics in primary rather than secondary care was also a key factor in encouraging participation. Patients suggested additional reassurance about the procedure would be helpful, particularly for those unsure about taking part.

The finding that invasive urodynamics is generally acceptable to patients supports quantitative studies [13, 14], which similarly report that most find the procedure as or better than expected. As found in previous qualitative research [8, 9], the interpersonal skills of nurses performing urodynamics, along with good explanation of the procedure, was key to patient acceptability. Building on previous work [8], which found that attending hospital to undergo urodynamics caused anxiety for older patients, we found interview participants preferred accessing the procedure in primary care. Findings also add to currently limited research on the effect of clinician-patient relationships on trial recruitment; as found in questionnaire studies [15, 16], patients’ familiarity with their GP appeared to influence their decision to participate.

This study provides an in-depth exploration of an under-researched area, adding new insights into patients’ acceptance of an invasive procedure in primary care. A key strength is our large and diverse sample, including the perspectives of primary healthcare professionals which have not previously been explored. Although we interviewed men across several geographical areas and of a wide range of ages, most were aged over 50. Therefore, findings may not be generalisable to younger men, who may experience urodynamic testing differently [17, 18]. Furthermore, we only interviewed three men who declined to take part in the urodynamic study due to difficulties recruiting participants from this group, so were unable to explore reasons for non-participation in detail. Some interviews were brief, dependent on the extent of participants’ involvement in the main PriMUS study; however, the research aims were highly focused and specific, enabling full examination of the research issue. Although our overall sample size is comparable to that obtained in similar qualitative studies (e.g. UPSTREAM [9]), a larger quantitative study based on this exploratory work would be useful to confirm findings. All interviewers were female, and none were qualified healthcare professionals, which may have affected the extent to which male patients were willing to discuss their experiences of urodynamic testing. However, interview participants appeared comfortable in disclosing detailed information about their experiences and feelings.

Study findings indicate several recommendations for future research and practice. Facilitating study procedures where possible in local primary care settings can result in increased participation rates and reduced anxiety for patients, particularly where the invitation to participate is from a familiar GP. More widely, conducting invasive urodynamics in primary care may encourage greater uptake of the procedure and reduce the need for hospital referral. Findings may also apply more widely to conducting urodynamics in community settings. If implementing this in practice, it would be important to ensure nurses performing urodynamics are comprehensively trained and have access to specialist advice. Having an experienced clinician to interpret urodynamic results and provide a clear, timely summary for GPs and patients is vital to ensure patients benefit fully from the invasive test. Dedicated space for urodynamics in a community hub setting would help overcome the logistical difficulties of transporting equipment and working in clinics with varying facilities. Due to the invasive nature of the urodynamic test, patients may benefit from additional reassurance (e.g. via telephone) prior to the procedure.

Future research could further explore the effects on study recruitment of performing research procedures in primary vs. secondary care settings and of patients being invited to participate by their own GP vs. other healthcare professionals. Greater exploration of the perspectives of patients who decline invasive urodynamics would provide a more rounded insight into acceptability of the procedure. Including the views of urodynamic nurses with a greater range of experience would also allow more in-depth exploration of the feasibility of conducting urodynamics in primary care.

## Conclusions

Findings indicate that conducting invasive urodynamics in primary care is feasible and acceptable to patients and healthcare professionals, and may encourage uptake of the procedure. Facilitating study procedures in familiar primary care settings can also impact positively on research recruitment. Expertise to help interpret urodynamic results, together with a support network for urodynamic nurses working remotely, is essential to ensure the potential benefits of the test are realised.

## Data Availability

The datasets generated and analysed during the current study are not publicly available and cannot be shared as individual privacy could be compromised if full interview transcripts were released.

## Abbreviations

LUTS: Lower urinary tract symptoms
GP: General practitioner
NIHR: National Institute for Health Research
HTA: Health Technology Assessment
PriMUS: Primary care management of lower urinary tract symptoms in men: development and validation of a diagnostic and clinical decision support tool
NICE: National Institute for Health and Care Excellence
MSP: Patients who participated in the main PriMUS study
IP: Patients who participated in the interview element of the PriMUS study only
UN: Urodynamic nurse
